# Hypermethylated TAGMe as a universal-cancer-only methylation marker and its application in diagnosis and recurrence monitoring of urothelial carcinoma

**DOI:** 10.1186/s12967-024-05420-3

**Published:** 2024-07-02

**Authors:** Zhicong Yang, Qing Chen, Shihua Dong, Peng Xu, Wanxiang Zheng, Zhanrui Mao, Chengchen Qian, Xiangyi Zheng, Lihe Dai, Chengyang Wang, Haoqing Shi, Jing Li, Jianlin Yuan, Wenqiang Yu, Chuanliang Xu

**Affiliations:** 1 Shanghai Public Health Clinical Center and Department of General Surgery, Huashan Hospital and Cancer Metastasis Institute and Laboratory of RNA Epigenetics, Institutes of Biomedical Sciences, Shanghai Medical College, Fudan University, Shanghai, China; 2 Department of Urology, Changhai Hospital, Naval Medical University, Shanghai, China; 3 Shanghai Epiprobe Biotechnology Co., Ltd, Shanghai, China; 4 Department of Urology, Xijing Hospital, Air Force Medical University, Xi’an, Shaanxi China; 5 Department of Bioinformatics, Center for Translational Medicine, Naval Medical University, Shanghai, China; 6 Department of Urology, Shanghai General Hospital, Shanghai Jiao Tong University School of Medicine, Shanghai, China

## Abstract

**Background:**

Urothelial carcinoma (UC) is the second most common urological malignancy. Despite numerous molecular markers have been evaluated during the past decades, no urothelial markers for diagnosis and recurrence monitoring have shown consistent clinical utility.

**Methods:**

The methylation level of tissue samples from public database and clinical collected were analyzed. Patients with UC and benign diseases of the urinary system (BUD) were enrolled to establish TAGMe (TAG of Methylation) assessment in a training cohort (n = 567) using restriction enzyme-based bisulfite-free qPCR. The performance of TAGMe assessment was further verified in the validation cohort (n = 198). Urine samples from 57 UC patients undergoing postoperative surveillance were collected monthly for six months after surgery to assess the TAGMe methylation.

**Results:**

We identified TAGMe as a potentially novel Universal-Cancer-Only Methylation (UCOM) marker was hypermethylated in multi-type cancers and investigated its application in UC. Restriction enzyme-based bisulfite-free qPCR was used for detection, and the results of which were consistent with gold standard pyrosequencing. Importantly, hypermethylated TAGMe showed excellent sensitivity of 88.9% (95% CI: 81.4–94.1%) and specificity of 90.0% (95% CI: 81.9–95.3%) in efficiently distinguishing UC from BUD patients in urine and also performed well in different clinical scenarios of UC. Moreover, the abnormality of TAGMe as an indicator of recurrence might precede clinical recurrence by three months to one year, which provided an invaluable time window for timely and effective intervention to prevent UC upstaging.

**Conclusion:**

TAGMe assessment based on a novel single target in urine is effective and easy to perform in UC diagnosis and recurrence monitoring, which may reduce the burden of cystoscopy.

*Trial registration* ChiCTR2100052507. Registered on 30 October 2021

**Supplementary Information:**

The online version contains supplementary material available at 10.1186/s12967-024-05420-3.

## Background

Early diagnosis of cancer has been an area of high-profile research. Emerging cancer detection technologies based on more accurate biomedical imaging [1–4], DNA mutation [5], epigenetic abnormality [6, 7], or metabolic products [8] have been developed and evaluated. Of these, DNA methylation aberration of specific genes occurs during the cancerous initiation and multiple progression stages, which has been considered as promising targets for the development of powerful diagnostic, prognostic, and predictive biomarkers [9–11]. Typically, *P16* methylation is identified as an early predictor for cancer progression from oral epithelial dysplasia [12], while plasma methylated *SEPT9* is testified as a diagnostic determinant for colorectal cancer screening and is available commercially [13]. However, a universal biomarker derived from common features of cancers has been rarely explored. In our previous report, we provided the concept of Universal-Cancer-Only Methylation (UCOM) and identified hypermethylated *HIST1H4F* as the first UCOM marker [14]. The characterization of DNA methylation in cancer provides important clues for early detection and opportunities for its potential applications.

Urothelial carcinoma (UC) is the second most common malignancy of the genitourinary system globally [15]. Approximately 70% of UC are non-muscle invasive urothelial carcinoma (NMIUC), and 30% are muscle-invasive urothelial carcinoma (MIUC). Despite NMIUC usually has good outcome (the 10-year survival rate is 80%) after transurethral resection of bladder tumor (TURBT), patients diagnosed with early-stage UCs will undergo frequent and lifelong surveillance (1-year and 5-year recurrence rate of 15–61% and 31–78%, respectively) [16]. Cystoscopy is one of the most used methods for UC surveillance [17], though sensitive, the invasiveness, discomfort, and high cost are hindering its application. Although urine cytology is another widely implemented alternative, its sensitivity is far from satisfactory for the clinical practice [18]. Meticulous and stringent follow-up checkups with repeat cystoscopy and urine cytology also double patients’ physical pain and financial burdens [19]. In addition, multifocality of UC makes it extremely hard to manage. Difficulties in ascertaining complete tumor removal in a piecemeal manner and possibilities of tumor re-implantation by TURBT impede the oncological control of UC [20]. Compromised by the concern of possible bladder perforation, complete resection is technically challenging and surgical margins need to be accurately evaluated to ensure the trade-off [21, 22]. Therefore, efficient and convenient strategies for accurate surgical resection determination and continuous recurrence monitoring could greatly improve the outcome and compliance of patients.

Currently, there are also numerous studies on methylation markers for diagnosis and recurrence monitoring of UC. One of the well-studied DNA methylation biomarkers EpiCheck (utilizing a panel of 15 methylation markers) presented with reported sensitivities of 62.5–90% and specificities of 82.1–90.0% in bladder cancer [23]. OncoUrine, a urine test consisting of hotspot mutations of 17 genes and the methylation biomarker *ONECUT2*, had a sensitivity of 80% and a specificity of 91.9% for the diagnosis of bladder cancer [24]. Despite the detection performance has been improved by combining multiple DNA methylation markers/DNA mutation sites, problems such as the lack of validation of large samples, complex detection methods, and high cost increase the difficulty of large-scale clinical translation [25, 26].

Herein, we identified a cancer cell-differentially methylated region (CC-DMR), located in the intergenic region in chr3: 5,026,052–5,027,247 (GRCh38/hg38), was significantly hypermethylated in multiple types of cancer. We defined this novel UCOM marker as TAGMe (TAG of Methylation) and further interrogated its application in UC. Results suggested that hypermethylated TAGMe presented excellent performance to distinguish UC from benign diseases of the urinary system (BUD) samples in a noninvasive and low-cost way, and showed promising potential in recurrence monitoring, thereby contributes to timely and effective intervention at an early stage.

## Methods

### TCGA and GEO DNA methylation data analysis

We performed an online PubMed search up to 1990 to obtain references for DNA methylation of cancer. The terms used for the PubMed search included DNA methylation, cancer, urothelial carcinoma, bladder, detection. A comparison was made with relevant articles to investigate the methylation level and differences of detection in UC. Methylation data of Illumina HumanMethylation450 Array/BeadChip and the Illumina Infinium EPIC arrays were derived from The Cancer Genome Atlas (TCGA) and Gene Expression Omnibus (GEO) database. The data were analyzed for each cancer type when both normal and cancer samples exceeded five. The absolute methylation level of TAGMe was calculated within an intergenic CpG island in chromosome 3 (chr3: 5,026,052–5,027,247, GRCh38/hg38). Detailed methylation levels of all UC samples used in TCGA and GEO database were listed in Additional file 1: Table S1.

### Clinical samples

In this study, all patients meeting all of the following inclusion criteria were recruited from Shanghai Changhai hospital between March 2022 and August 2023: (1) males or females aged over 18 years old and were willing to provide 30–50 ml of urine samples; (2) patients diagnosed with BUD without tumor history or patients were suspected with UC and planned to undergo surgery or urological endoscopy for this study. All enrolled participants signed an informed consent form. Urine samples were blinded when being transferred to laboratory personnel for methylation detection. Next, patients with one of following criteria were excluded: (1) samples with failed quality control (DNA content of urine samples less than 100 ng); (2) failed assay. Two months after the last patient was enrolled, the sample information was unblinded. Patients meeting any of the following criteria were excluded: (1) lost clinically pathological confirmation; (2) pathologically determined to be any other malignancies, such as kidney cancer and prostate cancer; (3) had a history of other types of cancer. Finally, methylation results and clinical information from eligible patients were included in the analysis. The above BUD controls consisted of patients diagnosed with urinary stones, cystitis glandularis, benign prostatic hypertrophy, and other benign diseases of the urinary system. Detailed demographic data of the subjects, including gender, age, etc. were listed in Additional file 1: Table. S2. Moreover, all clinical tissue samples were collected from Fudan University Shanghai Cancer Center.

### DNA extraction

DNA extraction from frozen tissue samples was conducted by the QIAGEN DNA extraction kit (Qiagen, 51306 and 56404). Genomic DNA extraction kit (Epiprobe Biotech, A-02) was used to conduct DNA extraction from urine samples. A total of 30–50 ml of urine was collected with Urine Collection Tube (Epiprobe Biotech, K-32). In accordance with the manufacturer’s instructions, the urine sample was centrifuged at 1000 g for 10 min to extract genomic DNA.

### Bisulfite-PCR pyrosequencing and DNA methylation evaluation

Bisulfite conversion was performed on a total of 100–200 ng of genomic DNA using the EZ DNA Methylation-Gold kit (ZYMO Research, D5006) according to the manufacturer's instructions. Approximately 50 ng of recovered bisulfite-treated DNA was used for subsequent PCR amplification. Amplification was performed using the *Taq* 2 × Master Mix Kit (NEB, M0270L). The PCR program was set to pre-denaturation at 98 °C for 30 s, followed by 45 cycles of amplification (98 °C for 10 s, 58 °C for 30 s, 72 °C for 30 s), and finally extension at 72 °C for 3 min. The amplified PCR product was confirmed by 2% agarose gel electrophoresis and pyro-sequencing using PyroMark™ Q24 ID (Qiagen). The methylation level was calculated as the average methylation level of CpG sites contained. For methylation standard curve and limit of detection (LOD) determination, genomic DNA (gDNA) from bladder cancer cell line T24 was used as positive control and urothelial cell from the urine of healthy volunteer (hereafter referred to as normal urothelial cell) was chosen as negative control.

### TAGMe methylation assessment

TAGMe methylation was performed by specialized laboratory researchers who were masked to the results of clinical diagnoses until DNA methylation detection was completed. The methylation level of TAGMe in urine samples was quantitatively detected by methylation sensitive restriction enzyme qPCR (MSRE-qPCR) as described previously [27, 28], and *GAPDH* gene was designed for normalization. Multiplex quantitative real-time PCR was performed on ABI 7500 Real-Time PCR System (Applied Biosystems, Inc) with a program of initiation at 95 °C for 10 min and 45 cycles of amplification (94 °C for 20 s and 60 °C for 60 s). The TaqMan probe and primers were as follows: forward primer: 5′-TGGGGCCTGCACCCTAGA-3′, reverse primer: 5′-AGGAGACCAAGAGCATCCCG-3′; and probe: 5′-TTCCTGAGTGGGCCGTGC-3′. DNA methylation level was calculated with normalization to *GAPDH*, as described in the following formula: ΔCt _(TAGMe methylation level)_ = Ct_TAGMe_ − Ct_*GAPDH*_. The formula of calculating the TAGMe value was determined using binary logistic regression analyzed by IBM SPSS Statistics software (20.0) and as follows, TAGMe Value = − 1.1 × ΔCt _(TAGMe methylation level)_ + 5.4. Taking the clinical diagnoses as the standard, the overall diagnostic accuracy was reflected by the area under the ROC curve. Youden index was used to determine the cutoff value, and cutoff was chosen when the Youden index is maximized.

### HE staining and pathological diagnosis

Surgical sections of UC were fixed with 4% paraformaldehyde for 1 h, embedded in paraffin, and then cross-sectioned to slices of 4 μm thickness. The obtained slices were then stained by Hematoxylin and Eosin Staining Kit (Beyotime, C0105S). The percentage of cancer cells in each slice was assessed semi-quantitatively by three experienced pathologists independently. Note that cancer-foci samples were taken from tumor foci, cancer-margin samples were taken from the surgical margin closest to the tumor (1–3 cm), and cancer-distal samples were taken from the most distal para-cancerous tissues.

### Statistics

ROC curve was analyzed using the hybrid Wilson/Brown method. Differences between two groups were analyzed by two-tailed unpaired Student’s t-test/nonparametric Mann–Whitney-Wilcoxon test, and one-way ANOVA followed by Dunnett’s multiple comparisons tests were performed when more than two groups were compared. Associations of urine test were evaluated, and correlations of first morning and three random time urine were determined using the Pearson correlation coefficient. A multivariable Cox regression model was constructed to evaluate the influence of several factors on recurrence-free survival (RFS) in UC patients. For each variable, hazard ratio (HR) with 95% confidence intervals was examined and statistical significance was accessed by *P* value. Kaplan–Meier analysis was employed to assess the effect of postoperative TAGMe value on RFS in UC patients. The log-rank test was utilized to compare the RFS of patients in the high and low TAGMe groups. All statistical analyses and data visualizations were carried out in R (3.6.0) with R packages and GraphPad Prism 9, and *P* value less than 0.05 was considered significant (*P < 0.05; **P < 0.01; ***P < 0.001; ****P < 0.0001).

## Results

### Hypermethylated TAGMe tracking as a novel UCOM marker

**Fig. 1:**
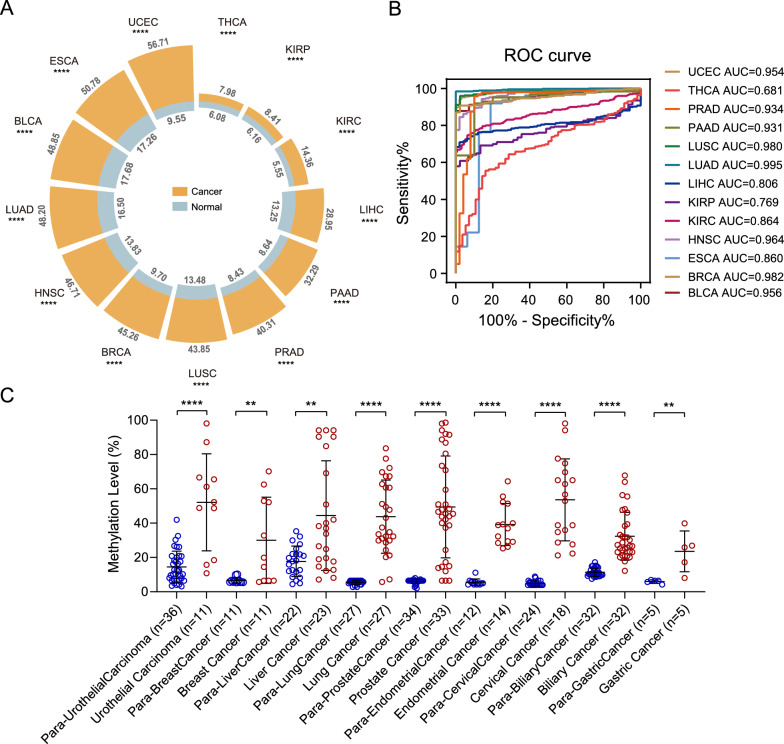
Hypermethylated TAGMe was identified as a UCOM marker. **A** Circular barplot showing the methylation level of TAGMe in 13 types of cancers from the TCGA database. BLCA, bladder urothelial carcinoma; BRCA, breast invasive carcinoma; ESCA, esophageal carcinoma; HNSC, head and neck squamous cell carcinoma; KIRC, kidney renal clear cell carcinoma; KIRP, kidney renal papillary cell carcinoma; LIHC, liver hepatocellular carcinoma; LUAD, lung adenocarcinoma; LUSC, lung squamous cell carcinoma; PAAD, pancreatic adenocarcinoma; PRAD, prostate adenocarcinoma; THCA, thyroid carcinoma; UCEC, uterine corpus endometrial carcinoma. The height of the bars represents the methylation level of cancer and corresponding normal samples, and the numbers labeled depict median methylation levels. **B** The AUC values for distinguishing cancer from para-cancer tissues across 13 types of cancers in TCGA. **C** Methylation level of TAGMe was further validated in clinical samples across nine types of cancer and para-cancer tissues. Scatter dot plot shows the mean ± SD. *P* values were calculated using the two-tailed nonparametric Mann–Whitney test. **P < 0.01; ****P < 0.0001

Whole-genome bisulfite sequencing (WGBS) has been widely used to quantify cytosine DNA methylation frequency. However, a built-in limitation of the bisulfite sequencing ultimately leads to the difficulties in aligning of bioinformatics analysis [29]. To solve the problem of low alignment rate in traditional DNA methylation sequencing methods, our previous work developed a novel approach, Guide Positioning Sequencing (GPS) for genome-wide DNA methylation detection, significantly improving the mapping accuracy [30]. Benefit from the GPS method, we identified many CC-DMR, based on which we suggested the concept of UCOM and applied it to the early cancer detection [14, 31, 32]. Different from past studies focused on the effects of hypermethylation of single gene’s specific promoter region, herein we noticed another CC-DMR located in the intergenic region in chr3: 5,026,052–5,027,247 (GRCh38/hg38), which we defined as TAGMe. Through extensive data mining, we identified that TAGMe as a potential UCOM marker was significantly hypermethylated in 13 types of cancers (Fig. 1A) with the area under the curve (AUC) of 11 types of cancers larger than 0.80 (Fig. 1B). To verify these analysis results, we evaluated TAGMe methylation of nine cancer types in collected clinical samples. TAGMe was hypermethylated in all nine cancer types compared to the para-cancer samples (Fig. 1C). Considering this unique feature, we identified hypermethylated TAGMe as a novel UCOM.

### TAGMe methylation exhibits applicational potential in UC detection

**Fig. 2:**
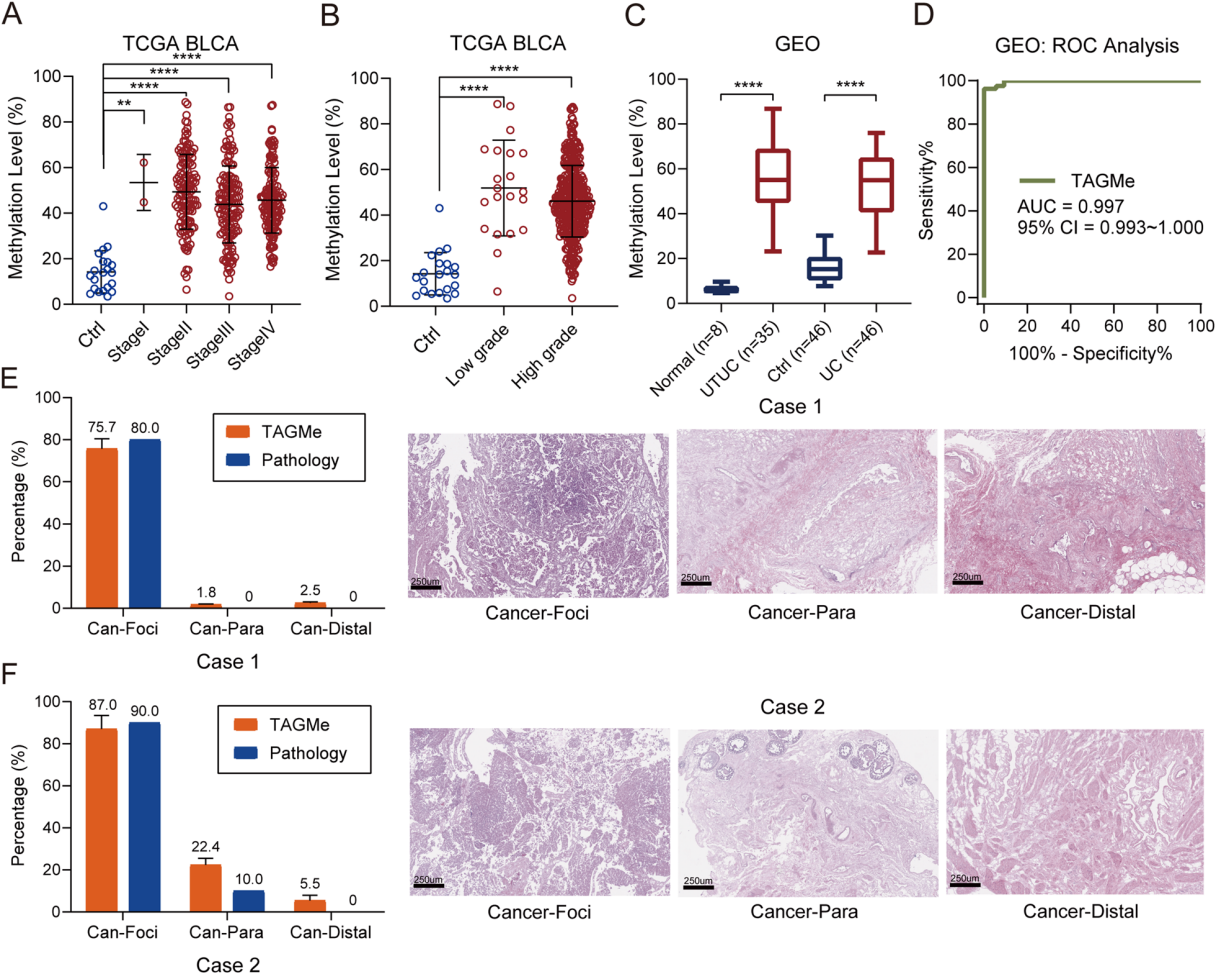
TAGMe was hypermethylated in UC. **A** TAGMe methylation in control and different stages of UC samples from the TCGA database. **B** Methylation level of TAGMe in TCGA BLCA cohort samples stratified by grade. Scatter dot plot shows the mean ± SD. **C** TAGMe hypermethylation was confirmed in UC samples from GEO database. The boxes represent the median ± 1 quartile, with the whiskers extending from the hinge to the smallest or largest value. **D** ROC curve and the associated AUC value of TAGMe in GEO datasets. **E**, **F** TAGMe methylation was evaluated by bisulfite-PCR pyrosequencing in paired Cancer-Foci, Cancer-Margin, and Cancer-Distal tissues in Case1 (**E**) and Case2 (**F**). Pathology was evaluated as proportions of cancer based on pathologists analyzed Hematoxylin–eosin (HE)-stained sections (left). HE staining of Cancer-Foci, Cancer-Margin, and Cancer-Distal tissues from UC patients who underwent a surgery (right). Data are presented by mean ± SD

Among the 13 types of cancers, UC sparked our special attention. Considering the unsatisfied sensitivity of urine cytology and invasive cystoscopy/ureteroscopy plus pathological biopsy, we would like to interrogate the applicability of TAGMe in UC. Firstly, TAGMe exhibited significant hypermethylation in UC when compared with controls in TCGA BLCA (bladder urothelial carcinoma) cohort (Fig. 2A and Additional file 1: Table. S1). Not only that, TAGMe has already been hypermethylated in the early stage of UC (Fig. 2A). Moreover, both low and high-grade UC were hypermethylated (Fig. 2B). We further conducted validation in GEO datasets, in which indicative TAGMe hypermethylation was also observed in UC (Fig. 2C and Additional file 1: Table S1). The ROC analysis showed both the AUC in TCGA and GEO datasets were above 0.9 (Figs. 1B, 2D). Collectively, these results supported the applicational potential of hypermethylated TAGMe in UC detection.

Pathological diagnosis based on morphology is the “gold standard” for diagnosis of UC. In recent years, studies have suggested that molecular mutations could be used as markers for early detection of UC [33–35]. We speculate that epigenetic variation will precede pathological changes, so it can be used as an indicator for early tumor detection. In order to verify this hypothesis, we collected urothelial cancer foci tissues (cancer-foci), paired proximal para-cancerous tissues (cancer-para, which may contain tumor residues), and cancer distal para-cancerous tissues (cancer-distal, no tumor residues). Firstly, for these samples, paraffin-embedding and HE staining were performed to evaluate the proportion of tumor components based on pathomorphology. Then, nucleic acid extraction was performed on the same slices, and the absolute methylation level of TAGMe was detected by the bisulfite-PCR pyrosequencing. Results showed that methylation levels of TAGMe were broadly concordant with the tumor components judged by pathologist in cancer-foci and cancer-distal samples, which exhibits abnormal or normal simultaneously. As for cancer-margin samples, hypomethylation of TAGMe basically matched with pathological diagnosis (Fig. 2E, F). However, Case4 patient with partially abnormal TAGMe methylation was assessed as negative by pathologist, which implied the precancerous state of cancer-margin and the potential risk of recurrence considering the occurrence timing of DNA hypermethylation preceded pathological phenotype changes (Additional file 2: Fig. S1B) [36]. These results indicated that TAGMe methylation can be used as an indicator of early detection of UC.

### Bisulfite-free TAGMe methylation detection with high sensitivity and stability

**Fig. 3:**
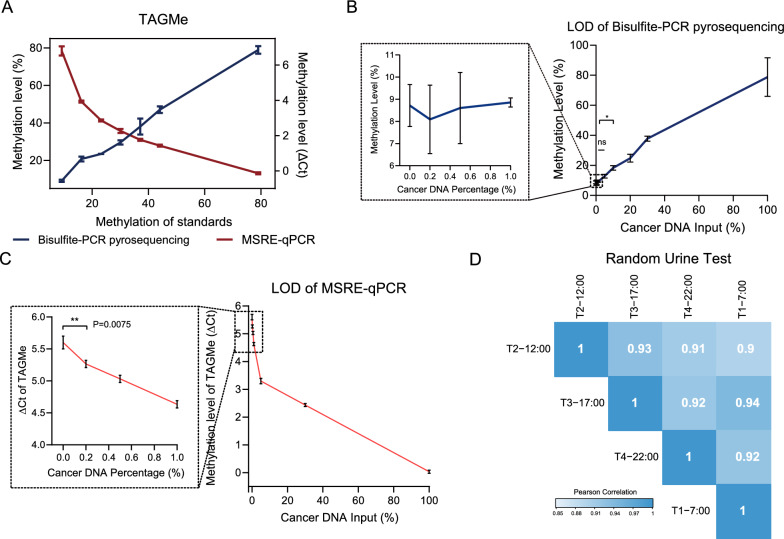
Sensitive and reliable methodology. **A** Performance of bisulfite-PCR pyrosequencing and MSRE-qPCR in detecting selected seven DNA methylation standard samples in the TAGMe genomic locus. The *x*-axis depicts the DNA methylation level; the *y*-axis in the left depicts the methylation level detected by bisulfite-PCR pyrosequencing, and the *y*-axis in the right depicts the ΔCt detected by MSRE-qPCR (ΔCt value reflects the DNA methylation level, and the higher value of ΔCt corresponds to the lower methylation level). The repeats of pyrosequencing and MSRE-qPCR were two and three for each grad, respectively. **B** LOD determination of TAGMe by bisulfite-PCR pyrosequencing. **C** Determination of the LOD by MSRE-qPCR. The mean ± SD values were plotted. **D** Heatmap summarizing urine test associations among the first morning (T1-7:00) and three random time (T2-12:00, T3-17:00, T4-22:00) samples in 15 enrolled patients. Pearson correlation coefficients were labeled. *P* values were calculated using the two-tailed unpaired Student’s t-test. *P < 0.05; **P < 0.01; ns, not significant

To address the problems of time-consuming and unstable to facilitate in clinic of bisulfite-PCR pyrosequencing, we performed MSRE-qPCR approach [31] based on methylation-sensitive restriction enzymes quantification to assess the methylation level of TAGMe. Bisulfite-free TAGMe methylation detection revealed consistent results to bisulfite-PCR pyrosequencing. The higher the calculated ΔCt value, the lower the methylation state, which drops dramatically in samples with low methylation levels (10–20%) (Fig. 3A). Additionally, we detected the limit of detection (LOD) by both bisulfite-PCR pyrosequencing and MSRE-qPCR, where 0.2% of the cancerous DNA components were stably detected by MSRE-qPCR (Fig. 3C), whereas bisulfite-PCR pyrosequencing was often undetectable when the cancerous DNA was less than 5% (Fig. 3B). This indicated that MSRE-qPCR can better meet the needs of sensitive, rapid, and easy-to-perform detection in clinic. Furthermore, multiple urine samples, including the first morning (T1-7:00) and three random time (T2-12:00, T3-17:00, and T4-22:00), were collected from subjects to eliminate confounding variables and confirm the validity of TAGMe. A high concordance among the four timepoints of urine was observed (Fig. 3D), suggesting the stability of TAGMe evaluation and random urine was also suitable for methylation detection.

### TAGMe hypermethylation enables efficient UC detection in urine

**Fig. 4:**
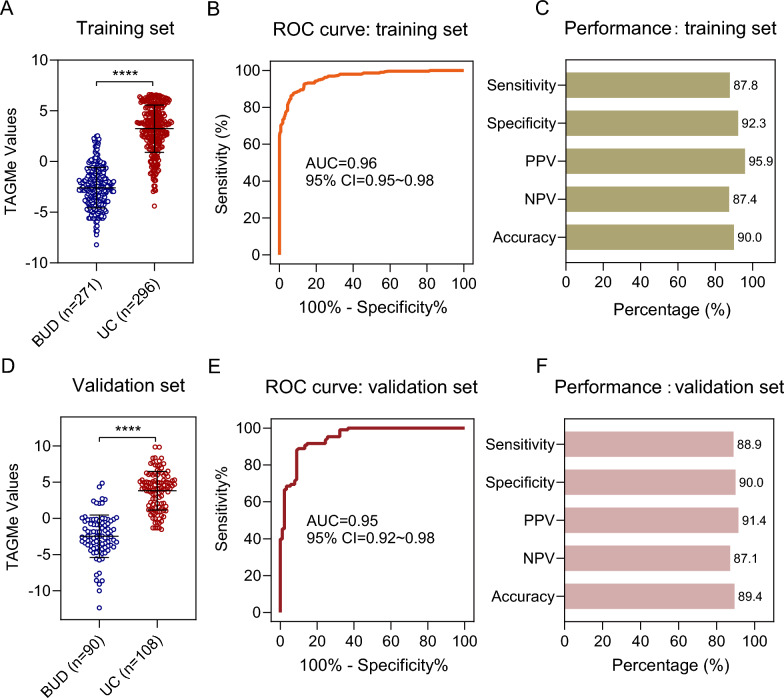
Performance of TAGMe assessment in urine for UC detection. **A** TAGMe value calculated by binary logistic regression in the training set. The higher TAGMe value represents a higher methylation status. **B** ROC curve analysis of TAGMe in the training set. **C** Performance of TAGMe in the training set. **D** TAGMe methylation was detected in the validation set. **E** ROC curve analysis of TAGMe in the validation set. **F** The sensitivity, specificity, accuracy, PPV, and NPV of TAGMe assessment in the validation set. Scatter dot plot shows the mean ± SD. *P* values were calculated using the two-tailed nonparametric Mann–Whitney test. ****P < 0.0001

To investigate the practicability of TAGMe in UC noninvasive detection, we collected urine for the establishment of TAGMe assessment. The enrollment of participants is summarized in Additional file 2: Fig. S2. A prospective blinded cohort was enrolled as training set to measure the performance of TAGMe assessment in urine. A total of 567 cases including 271 BUD and 296 UC were enrolled from Shanghai Changhai Hospital (Additional file 1: Table S2). TAGMe value was calculated to reflect the methylation status and the higher TAGMe value represented a higher methylation status. It turned out that UC group showed higher level of TAGMe value (Fig. 4A). ROC analysis showed high AUC of 0.96 (95% CI: 0.95–0.98) (Fig. 4B), which could effectively distinguish UC from BUD patients with the sensitivity of 87.8% (95% CI: 83.6–91.3%) and specificity of 92.3% (95% CI: 88.4–95.1%) (Fig. 4C). Further, another independent blinded cohort was recruited as validation set to verify the performance of TAGMe assessment. A total of 198 urine samples were collected, including 90 BUD samples and 108 UC samples. Accordingly, prominent higher level of TAGMe values was also observed in UC (Fig. 4D) and AUC of ROC curve was 0.95 (95% CI: 0.92–0.98) (Fig. 4E). TAGMe exhibited satisfied performance to distinguish UC from BUD with the sensitivity of 88.9% (95% CI: 81.4–94.1%), the specificity of 90.0% (95% CI: 81.9–95.3%), a PPV of 91.4% (95% CI: 84.4–96.0%), a NPV of 87.1% (95% CI: 78.6–93.2%), and the overall accuracy rate of 89.4% (95% CI: 84.3–93.3%) in the validation set (Fig. 4F). The high consistency between the training and validation set suggested TAGMe can efficiently distinguish BUD from UC samples in a noninvasive manner.

Detailed, TAGMe assessment showed a high sensitivity of 85.4% for most commonly NMIUC and a sensitivity of 92.3% for MIUC. About one-third of all bladder cancers occur as a multifocal disease forming several tumors simultaneously at different sites of the bladder wall. Herein, TAGMe showed equally high sensitivity of 87% for single-focal and multi-focal UC. Even for tumors less than 1 cm in diameter, the sensitivity of TAGMe assessment is as high as 84.2% (Table 1). These results indicated that hypermethylated TAGMe performed well in different clinical scenarios of UC.

**Table 1:** The performance of TAGMe assessment stratified by different clinical characteristics

Clinical characteristics	TAGMe positive	Urothelial Carcinoma	Sensitivity (95% CI)
Invasiveness			
NMIUC	211	247	85.4% (80.4–89.6%)
MIUC	120	130	92.3% (86.3–96.3%)
Grade			
Low grade	75	97	77.3% (67.7–85.2%)
High grade	231	253	91.3% (87.1–94.5%)
Tumor numbers			
Single	149	171	87.1% (81.2–91.8%)
Multiple	129	148	87.2% (80.7–92.1%)
Tumor size			
< 1 cm	32	38	84.2% (68.8–94.0%)
1–3 cm	145	169	85.8% (79.6–90.7%)
> 3 cm	92	102	90.2% (82.7–95.2%)

### TAGMe hypermethylation for UC recurrence surveillance

**Fig. 5:**
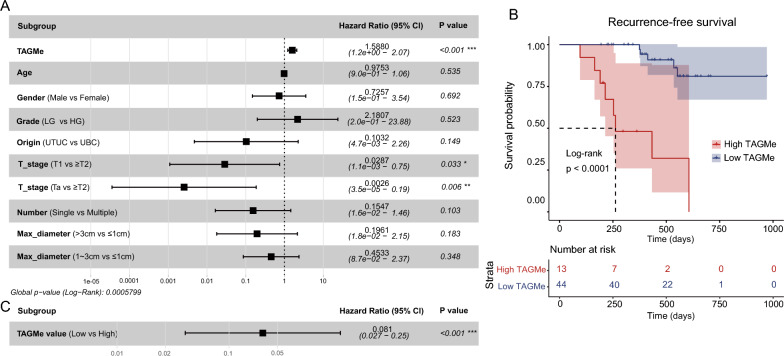
TAGMe hypermethylation was used for UC recurrence monitoring. **A** Forest plot shows the result of multivariate Cox regression model of postoperative UC patients. CI, confidence interval; TAGMe, mean monthly postoperative TAGMe value after surgery; Number, number of tumor foci; Max_diameter, max diameter of tumor. **B** Kaplan–Meier analysis of Recurrence-free survival (RFS) stratified by TAGMe. *P* value was evaluated using the log-rank test. Patients with mean postoperative TAGMe level above the cutoff were classified as the high TAGMe group (13/57 cases) and the remaining patients were categorized into the low TAGMe group (44/57 cases). **C** Forest plot shows the result of univariant Cox regression model of postoperative TAGMe assessment

Given the susceptibility of UCs relapse, long-term invasive surveillance is usually required for patients. Thus, we wonder that whether TAGMe assessment could be used for recurrence monitoring. We firstly enrolled 57 UC patients undergoing postoperative surveillance, and urine samples were collected monthly for six months after surgery to assess the TAGMe methylation. Detailed clinical characteristics of these patients are presented in Additional file 1: Table S3. Recurrence was defined as biopsy-proven cancer or strong alternative imaging evidence of relapse/metastasis. We examined several potential exposure variables including postoperative TAGMe assessment, gender, grade, age, tumor stage, number of tumor foci, and max diameter of tumor by multivariable Cox regression analysis and ultimately identified that postoperative TAGMe assessment was an independent risk factor for RFS in UC patients (*P* < 0.0001) (Fig. 5A). Subsequently, patients were classified as the high methylation group (13/57 cases) and low methylation group (44/57 cases) according to the cutoff of TAGMe. Kaplan–Meier and univariate Cox regression analysis showed that the mean postoperative TAGMe values were negatively correlated with the RFS of UC patients (*P* < 0.001) (Fig. 5B). Individuals with higher levels of postoperative TAGMe had a significantly increased risk of recurrence (HR: 0.081, 95% CI: 0.027–0.25, ***P < 0.001) (Fig. 5C), indicating that TAGMe also presented excellent efficiency in UC prognosis monitoring. Despite the recurrence of UC could be detected by cystoscopy eventually, it would be more meaningful if incomplete resections could be found earlier, by which timely intervention would be taken to prevent UC upstaging. Herein, we noticed that 9 out of 13 patients with high TAGMe values relapsed after a period of time when TAGMe was judged to be positive, and 8 of them had developed abnormal TAGMe values at the third month after surgery, which preceded clinical diagnosis by three months to one year (Additional file 1: Table. S4). In aggregate, these results further proved that hypermethylated TAGMe enabled earlier detection of recurrence and progression.

## Discussion

In the current study, we revealed that hypermethylated TAGMe functions as a UCOM with discernible value for UC diagnosis. We developed TAGMe assessment based on the restriction enzyme-based bisulfite-free qPCR with 90.0% of specificity and 88.9% of sensitivity. Furthermore, we suggested its potential application in the recurrence surveillance of UC. The abnormality of TAGMe preceded clinical recurrence by three months to one year, by which prompt intervention would be taken to prevent UC upstaging.

For traditional methylation detection such as WGBS and pyrosequencing, bisulfite conversion leads to 84–96% DNA degradation, which reduces the number of DNA molecules that can be effectively analyzed [37]. We optimized the assay for the detection of DNA methylation using enzyme-based bisulfite-free qPCR to minimize the loss of DNA. Accordingly, this assay is more sensitive, stable, and reproducible. Clinical samples were included for double-blind validation, and the overall compliance rate of urine samples reached 89%. Importantly, the detection is quick (finalized in 2–3 h), low-cost, practicable (only qPCR instrument needed), and easily automated (standardized operations and procedures), which has a huge market for regions with strained medical resources.

Although TURBT can be used to manage grossly visible lesions, for many patients, the degree of progression of the tumor is beyond the grasp of the surgeon [38]. Studies of enhanced cystoscopy showed that more than 20% of non-muscle invasive bladder cancer (NMIBC) could not be detected by standard procedures [39]. Evidences from radical cystectomy specimens suggested that the urothelium with morphologically grossly normal specimens harbors molecular genetic changes similar to those of the primary tumor [40]. Additionally, the status of surgical margins not only influences the likelihood of local recurrence but also correlates with the progression of the disease and disease-specific mortality in patients underwent radical cystectomy [41–43]. It is noteworthy that TAGMe evaluation was more objective than pathological assessment. Based on this, a rapid detection method can be developed to improve the completeness of tumor resection.

In spite of proper management, 50–70% of NMIUC will recur, and 10–15% of these may progress to muscular aggressive disease [44–46]. According to the AUA/SUO guidelines, the surveillance protocol for high-risk NMIUC recommends cystoscopy and cytology every 3–6 months for two years, then 6–12 months for years three and four, and then annually thereafter [47], which brings substantial cost and pain incurred to patients. TAGMe assessment as a noninvasive and effective assay could detect cancer lesions earlier. Patients only need to provide urine to monitor the dynamic changes of TAGMe instead of undergoing frequent invasive cystoscopy, and physicians need to pay special attention to patients with abnormal TAGMe to ensure follow-up. This would greatly simplify the difficulty of continuous monitoring and reduce overly intense cystoscopy follow-up. Additionally, TAGMe assessment combined with imaging is expected to replace invasive cystoscopy and biopsy (the gold standard) before TURBT. For postoperative assessment of minimal residual disease (MRD) and recurrence monitoring, TAGMe detection could be an alternative/supplement to appropriately extend the interval between postoperative cystoscopy and reduce unnecessary cystoscopies [48]. Despite the merits mentioned above, the current study has some limitations that need to be improved in the future. This study was conducted in a single center, and further validation of multiple centers was lacking. The number of patients enrolled for recurrence surveillance is limited. A larger sample size and a long-term follow-up observation are needed to further validate the clinical feasibility of TAGMe evaluation in recurrence monitoring.

## Conclusion

Taken together, our study uncovered that TAGMe assessment conducted by bisulfite-free qPCR in urine emerges as a noninvasive and effective approach for UC diagnosis and recurrence surveillance. Expectantly, its implementation is anticipated to improve UC continuous management to fewer avoidable cystoscopies and associated costs.

### Supplementary Information

**Additional file 1: Table S1.** Detailed methylation level of TAGMe in TCGA and GEO datasets. **Table S2.** Clinical characteristics of the training and validation set. **Table S3.** Clinical characteristics of the recurrence monitoring set. **Table S4.** Detailed information of patients in the postoperative high TAGMe group.

**Additional file 2: Figure S1.** Application of TAGMe for UC residual evaluation. **Figure S2.** Workflow of the study design.

## Data Availability

The data generated or analyzed during this study are included in this manuscript and its additional files.

## Abbreviations

AUC: Area under the curve
BLCA: Bladder urothelial carcinoma
BUD: Benign urological disease
GEO: Gene expression omnibus
KM: Kaplan–Meier
LOD: Limit of detection
MIUC: Muscle invasive urothelial carcinomas
MRD: Minimal residual disease
MSRE-qPCR: Methylation sensitive restriction enzyme qPCR
NPV: Negative predictive value
NMIUC: Non-muscle invasive urothelial carcinomas
PPV: Positive predictive value
QC: Quality control
RFS: Recurrence free survival
ROC: Receiver operating characteristic
TAGMe: TAG of methylation
TCGA: The Cancer Genome Atlas
UC: Urothelial carcinoma
UCOM: Universal cancer only methylation
