# Supplementary material for: Hypermethylated TAGMe as a universal-cancer-only methylation marker and its application in diagnosis and recurrence monitoring of urothelial carcinoma
Source: J Transl Med. 2024 Jul 2;22:608. doi: 10.1186/s12967-024-05420-3 (PMC11218302; doi:10.1186/s12967-024-05420-3)
Supplement: Supplementary file 2 — Additional file 2: Figure S1. Application of TAGMe for UC residual evaluation. Figure S2. Workflow of the study design. [file 12967_2024_5420_MOESM2_ESM.docx]

**Hypermethylated TAGMe as a Universal-Cancer-Only Methylation marker and its application in diagnosis and recurrence monitoring of** **urothelial carcinoma**

Zhicong Yang^1†^, Qing Chen^2†^, Shihua Dong^1,3†^, Peng Xu^1,3†^, Wanxiang Zheng^4^, Zhanrui Mao^1^, Chengchen Qian^3^, Xiangyi Zheng^3^, Lihe Dai^2^, Chengyang Wang^3^, Haoqing Shi^2^, Jing Li^5^, Jianlin Yuan^4*^, Wenqiang Yu^1*^, and Chuanliang Xu^2,6**^


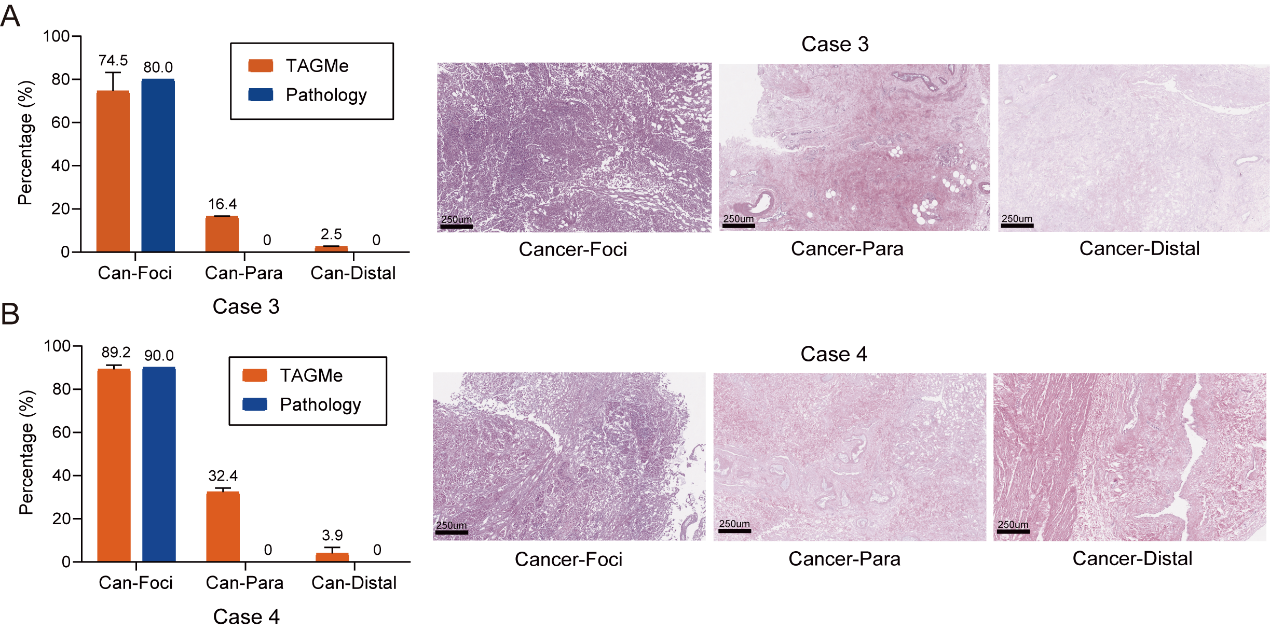


**Supplementary Figure 1.** Application of TAGMe for UC residual evaluation. (A-B) TAGMe methylation detected by bisulfite-PCR pyrosequencing in pared Cancer-Foci, Cancer-Margin, and Cancer-Distal tissues of Case3 (A) and Case4 (B). Pathology was evaluated as proportions of cancer based on pathologists analyzed Hematoxylin-eosin (HE)-stained sections (left). HE staining of Cancer-Foci, Cancer-Margin, and Cancer-Distal tissues from UC patients (Case3 and Case4) who underwent a surgery (right). Data are presented by mean ± SD.


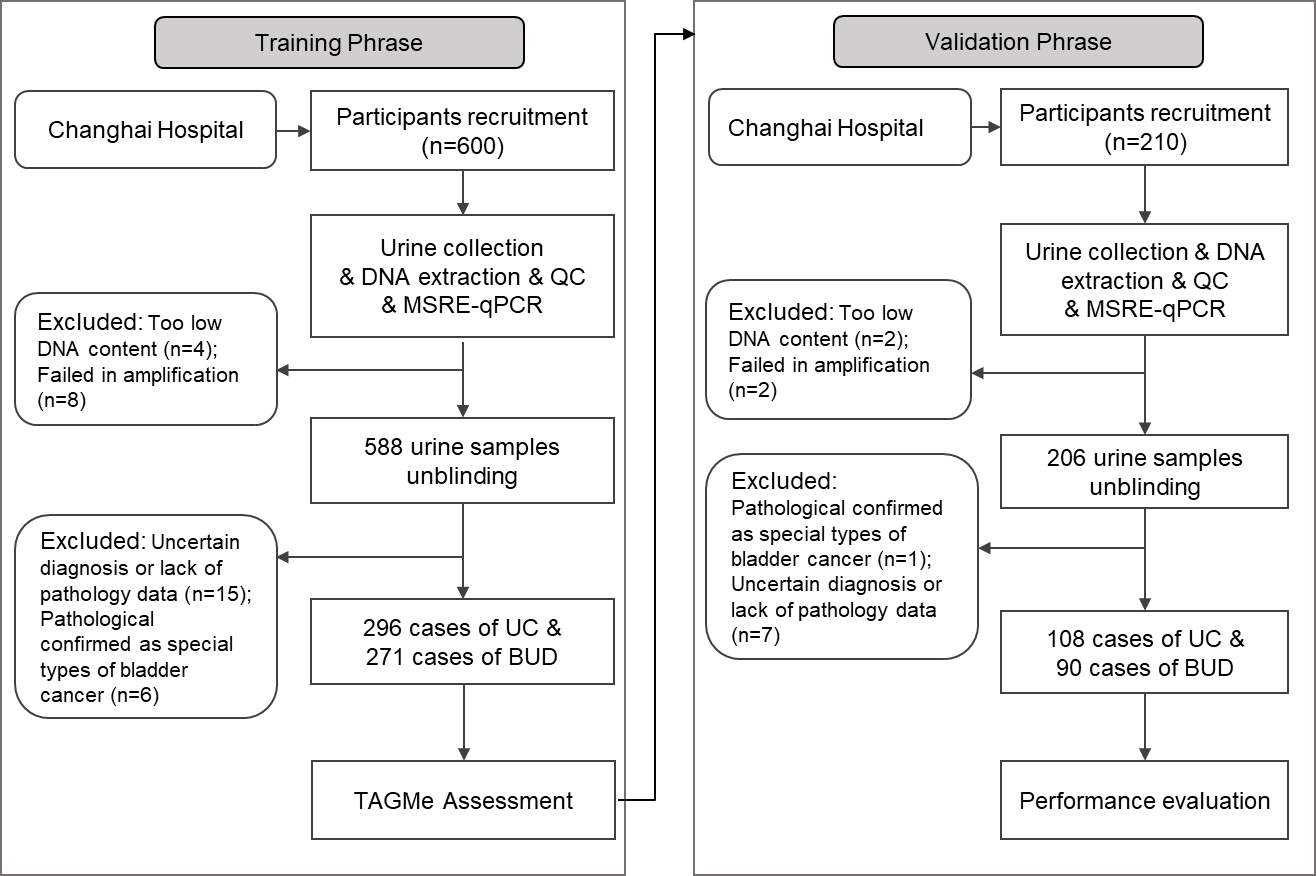


**Supplementary Figure 2.** Workflow of the study design. BUD, benign urological disease; MSRE-qPCR: methylation sensitive restriction enzyme qPCR; QC, quality control; UC, urothelial carcinoma.
